# Epidemiology and outcomes of people with dementia, delirium, and unspecified cognitive impairment in the general hospital: prospective cohort study of 10,014 admissions

**DOI:** 10.1186/s12916-017-0899-0

**Published:** 2017-07-27

**Authors:** Emma L. Reynish, Simona M. Hapca, Nicosha De Souza, Vera Cvoro, Peter T. Donnan, Bruce Guthrie

**Affiliations:** 1 Dementia and Ageing Research Group, Faculty of Social Science, University of Stirling, Stirling, FK9 4LA UK; 2 Population Health Sciences Division, University of Dundee, Dundee, DD2 4BF UK; 3 NHS Fife, Fife, Kirkcaldy, KY2 5AH UK; 4 Epidemiology and Biostatistics, Population Health Sciences Division, University of Dundee, Dundee, DD2 4BF UK; 5 Primary Care Medicine, Population Health Sciences Division, University of Dundee, Dundee, DD2 4BF UK

**Keywords:** Dementia, Delirium, Cognitive impairment, Length of stay, Mortality, Readmission

## Abstract

**Background:**

Cognitive impairment of various kinds is common in older people admitted to hospital, but previous research has usually focused on single conditions in highly-selected groups and has rarely examined associations with outcomes. This study examined prevalence and outcomes of cognitive impairment in a large unselected cohort of people aged 65+ with an emergency medical admission.

**Methods:**

Between January 1, 2012, and June 30, 2013, admissions to a single general hospital acute medical unit aged 65+ underwent a structured specialist nurse assessment (n = 10,014). We defined ‘cognitive spectrum disorder’ (CSD) as any combination of delirium, known dementia, or Abbreviated Mental Test (AMT) score < 8/10. Routine data for length of stay (LOS), mortality, and readmission were linked to examine associations with outcomes.

**Results:**

A CSD was present in 38.5% of all patients admitted aged over 65, and in more than half of those aged over 85. Overall, 16.7% of older people admitted had delirium alone, 7.9% delirium superimposed on known dementia, 9.4% known dementia alone, and 4.5% unspecified cognitive impairment (AMT score < 8/10, no delirium, no known dementia). Of those with known dementia, 45.8% had delirium superimposed. Outcomes were worse in those with CSD compared to those without – LOS 25.0 vs. 11.8 days, 30-day mortality 13.6% vs. 9.0%, 1-year mortality 40.0% vs. 26.0%, 1-year death or readmission 62.4% vs. 51.5% (all *P* < 0.01). There was relatively little difference by CSD type, although people with delirium superimposed on dementia had the longest LOS, and people with dementia the worst mortality at 1 year.

**Conclusions:**

CSD is common in older inpatients and associated with considerably worse outcomes, with little variation between different types of CSD. Healthcare systems should systematically identify and develop care pathways for older people with CSD admitted as medical emergencies, and avoid only focusing on condition-specific pathways such as those for dementia or delirium alone.

**Electronic supplementary material:**

The online version of this article (doi:10.1186/s12916-017-0899-0) contains supplementary material, which is available to authorized users.

## Background

Ageing populations mean that health and social care systems internationally are increasingly stressed by large increases in the number of multi-morbid and frail people needing care who fit badly in systems designed to manage single conditions. People with dementia and other disorders resulting in confusion are an important subset of frail older people who present specific challenges, particularly when admitted to acute hospitals. In 2001, the UK Department of Health [1] estimated that two-thirds of hospital beds were occupied by patients aged over 65 years, up to half of whom might have some kind of cognitive impairment, including dementia and delirium [2]. In the US, people with cognitive impairment have more than three times as many hospital stays as individuals who are hospitalised for some other condition [3, 4]. Further, inpatient costs were found to be higher for both Medicare beneficiaries with Alzheimer’s disease and other dementias than for other Medicare beneficiaries and for inpatients with delirium compared to those without [5, 6].

In the hospital setting, cognitive impairment may be due to a number of overlapping conditions. People may have pre-existing dementia before admission, may develop delirium (characterised by an acute onset of confusion, a fluctuating course and inattention) as part of the acute illness precipitating admission, or may have delirium superimposed on dementia. Finally, unspecified cognitive impairment due to undiagnosed dementia or delirium, adverse effects of medication, poorly controlled physical morbidities (e.g. diabetes) or a combination of these is also common. The symptoms and presenting features of all these conditions show considerable overlap, which can lead to misdiagnosis; for example, the onset of neuropsychiatric symptoms in a patient with dementia may be labelled as worsening of their dementia rather than properly attributed to delirium. We therefore use the term ‘cognitive spectrum disorders’ (CSD) to signify the presence of cognitive impairment whether formally diagnosed or not.

Older people admitted to hospital with a CSD are a heterogeneous and highly vulnerable population who are typically poorly assessed and managed, and it is important to better understand their needs in order to focus care and treatment. However, most research in older people admitted to hospital has studied either dementia or delirium in isolation, and is most commonly done on relatively small cohorts of selected volunteers in specialist geriatric settings, risking selection bias and poor generalisability. Relatively few studies have examined outcomes in this population, particularly outcomes after discharge. Systematic reviews which separately examined dementia [7], delirium [8, 9] and delirium superimposed on dementia [4] in hospital inpatients have been published. Findings show the estimated prevalence in hospital inpatients varied from 9% to 63% for dementia [7], 10% to 31% for delirium [8], and 32% to 89% for dementia with superimposed delirium [10]. The estimated prevalence of cognitive impairment of any cause varied from 21% [11] to 40% [12]. Prevalence varied depending on the population studied (e.g. specialist settings vs. unselected medical admissions; early vs. later assessment after admission, age range considered) and the assessment methods used, with dementia assessment not normally including a delirium screen increasing the risk of misclassification.

The dementia review [7] included studies showing significant associations with increased length of stay (LOS), functional decline and discharge to institutional care, but there was no association with increased mortality in the one study examining this [13], and no study examined readmission [7]. A prospective cohort study performed after the review found that people with dementia had an increased risk of in-hospital death (adjusted HR 2.09, 95% CI 1.10–4.00) [14]. The delirium review found an increased risk of death at mean follow-up of 22.7 months (HR 1.95, 95% CI 1.51–2.52) [8]. More recently, studies of delirium superimposed on dementia have examined outcome. A number of these have suggested an association with higher mortality [15, 16]. Few studies of cognitive impairment in general reported associations with outcomes, but more severe cognitive impairment on admission was associated with both increased institutionalisation [17, 18] and mortality [18].

Unlike most previous research in selected volunteers, this study is based on structured assessment of all CSDs (dementia, delirium and cognitive impairment) in a large unselected population of people aged ≥ 65 years admitted as an acute medical emergency, with an assessment completed in 10,014 (79.0%) of admissions. The objective of this study was therefore to examine the relative frequencies and the associated outcomes of people with the full range of CSDs in a large, unselected population of older people admitted as an acute medical emergency.

## Methods

The design is a prospective cohort study of all people aged 65 years and over with an acute medical admission to one district general hospital in the Fife region of Scotland, with complete 1 year follow-up. This study reports data for all older people admitted to the acute medical unit (AMU) between January 2012 and June 2013 inclusive.

NHS Fife provides acute medical care from a single 640-bedded district general hospital to a diverse urban and rural population of ~360,000. Fife has an ageing population and has seen a 76% rise in emergency admissions of people aged 65 and over during the last 10 years. The population of Fife accounts for 7% of the population of Scotland and its acute healthcare system comprises the acute hospital in addition to four non-acute community hospitals. There is no other inpatient healthcare provision in Fife. In the period of the study, all emergency medical admissions from any source were admitted via a single AMU with subsequent discharge or step-down to appropriate medical wards after 12–24 hours. Medical admissions are unselected with exception to acute stroke and acute ST elevation myocardial infarction. Operative orthopaedic trauma patients are admitted via the Surgical Admissions Unit and all non-operative trauma patients are admitted via the Medical Admissions Unit. At the time of censoring, the Hospital at Home service was not yet operational and therefore alternatives to admission were not available.

Starting in 2009 and funded by the Scottish Government Joint Improvement Team, the NHS Fife Dementia Co-ordinating Group designed and implemented the Older Persons Routine Acute Assessment (OPRAA). OPRAA is based on the principles of “comprehensive geriatric assessment” [19], with trained specialist nurses carrying out a structured assessment during the first 24 hours of admission, including an Abbreviated Mental Test (AMT) [20], the Confusion Assessment Method (CAM) [21] for the presence of delirium, an assessment of the presence of delirium based on clinical history, examination and informant report, and documentation of the presence of a pre-admission diagnosis of dementia from self/informant report and/or hospital and primary care records. By design, individuals with a predicted LOS less than 24 hours, where death was expected, or with an acute illness requiring critical care intervention do not undergo an OPRAA.

All specialist nurses carrying out the assessment underwent a structured training programme for the use of these tools, delivered by a specialist mental health nurse and an occupational therapist. For the CAM, the specialist nurses underwent training in its use as set out in the training manual for CAM that was available in 2009. In addition to this, the findings of the paper by Inouye et al. [22], namely that nurses who based assessment of delirium on routine clinical observations “*often missed delirium when present, but rarely identified delirium when absent*” and that “*Recognition of delirium can be enhanced with education of nurses in delirium features, cognitive assessment, and factors associated with poor recognition*”, were taken into account and all nurses had training in cognitive assessment and in the features of delirium. Specialist nurse training began in autumn 2008, with initial implementation of OPRAA commencing in January 2009, meaning that, by the time of collection of the data analysed (January 2012 to June 2013), evaluation was being routinely performed by very experienced staff.

All people aged 65 years and older admitted to the AMU were identified from Scottish Morbidity Records 01 (SMR01) data, which is a validated NHS Scotland routine dataset including age, sex, date of admission and discharge, type of admission, and whether the patient was admitted from a residential care or nursing home. The SMR01 dataset was then linked to the Community Health Index (CHI – the NHS Scotland patient register), the OPRAA dataset, SMR04 data on psychiatric admissions, CHI national mortality data and community dispensed prescribing data, resulting in a linked dataset of all admissions between January 2012 and June 2013 inclusive, with at least 1 year of follow-up data for LOS, death and re-admission. OPRAA was used to define the presence of a CSD, defined as one or more of known dementia diagnosed before admission, delirium and unspecified cognitive impairment. Dementia was defined as a reported dementia diagnosis in the OPRAA assessment, a prior community prescription of drugs for dementia (anticholinesterase inhibitors or memantine), or a prior dementia diagnosis recorded in SMR01 or SMR04. Delirium was defined as both full syndromic delirium (a positive score on the CAM) or a clinical diagnosis of delirium made by the specialist nurses. Outcomes were defined as inpatient LOS, death within 30 days and 1 year after admission, and a composite of death or readmission within 30 days or 1 year from discharge (competing risks mean that we did not examine readmission alone, since those who die cannot be readmitted and post-discharge mortality varies between groups).

Data linkage used the CHI number (the NHS Scotland unique patient identifier) and was carried out by the University of Dundee Health Informatics Centre (HIC). HIC Standard Operating Procedures (SOPs) have been reviewed and approved by the NHS East of Scotland Research Ethics Service, which does not require review of individual projects provided they follow SOPs and obtain Caldicott permission to use the data. This project used HIC SOPs and consent for research using this data was obtained from the NHS Fife Caldicott Guardian, based on researcher access only to anonymised data held in the HIC ISO27001 and Scottish Government accredited safe haven [23, 24].

Data for all admissions over the 18 month period of January 2012 to June 2013 were included in the study. Characteristics of patients at the time of admission were defined as described above, for all admissions (admission cohort) and for an incident cohort of patients admitted (defined as being the first admission for an individual in the study period where there had not been a previous admission in the prior 6 months).

Comparative descriptive statistics based on means and proportions for patients with and without an OPRAA assessment were calculated, with 95% confidence intervals (CIs) for proportions or difference between two proportions calculated based on Wilson’s method [25, 26]. Logistic regression (binary and multinomial) was used to test for main and interaction effects of sex and age on prevalence of CSDs. For the missing variables of delirium and dementia, it was assumed that patients did not have the condition, whereas for missing AMT scores, it was assumed that patients did not have unspecified cognitive impairment (low AMT) only in absence of known dementia or delirium. Data linkage and analysis was carried out using SAS® 9.4 software (SAS Institute Inc., Cary, NC, USA).

## Results

### All admissions

Between January 2012 and June 2013, there were 12,673 admissions for 8374 individuals aged 65 years and over to the AMU, accounting for 61% of all AMU admissions. An OPRAA was completed in 10,014 (79%) admissions. Of those patients who did not undergo an OPRAA assessment (n = 1632), 1102 (67.52%) had a LOS ≤ 2 days, and 59 patients (3.62%) died within 5 days. Admissions where an OPRAA assessment was completed were for patients who were on average 2.3 (95% CI 2.0–2.7) years older than admissions without an OPRAA assessment, more commonly for women (56.7% vs. 51.4%, difference 5.3%, 95% CI 3.2–7.4), more likely to be admissions from a care home (8.0% vs. 5.8%, difference 2.1%, 95% CI 1.1–3.1), and less likely to have a short LOS (31.4% staying 0–2 days vs. 66.6%, difference 35.2%, 95% CI 33.2–37.2). The same patterns were present in the incident cohort (Table 1).

**Table 1:** Descriptive characteristics and prevalence of cognitive spectrum disorders for all emergency admissions for people aged ≥ 65 years

	All admissions – received an OPRAA assessment N = 10,014 (79.0% of all admissions)		All admissions – did not receive an OPRAA assessment N = 2659 (21.0% of all admissions)		Incident admissions – received an OPRAA assessment N = 5569 (77.3% of incident admissions)		Incident admissions – did not receive an OPRAA assessment N = 1632 (22.7% of incident admissions)	
Age (mean (95% CI)), years		79.3 (79.2–79.5)		77.0 (77.6–77.3)		79.1 (79.5–79.9)		76.2 (75.9–76.6)
Female	5679	56.7 (55.7–57.7)	1367	51.4 (49.5–53.3)	3121	56.0 (54.7–57.3)	850	52.1 (49.7–54.5)
Admitted from care home	799	8.0 (7.5–8.5)	155	5.8 (5.0–6.8)	382	6.9 (6.3–7.6)	76	4.7 (3.8–5.8)
Length of stay (mean days (95% CI))		16.8 (16.2–17.4)		7.4 (6.5–8.3)		16.5 (15.6–17.4)		6.7 (5.6–7.8)
0–2 days	3141	31.4 (30.5–32.3)	1770	66.6 (64.8–68.4)	1867	33.5 (32.3–34.8)	1145	70.2 (67.9–72.4)
3–5 days	1652	16.5 (15.8–17.2)	285	10.7 (9.6–11.9)	897	16.1 (15.2–17.1)	150	9.2 (7.9–10.7)
6–9 days	1503	15.0 (14.3–15.7)	180	6.8 (5.9–7.8)	797	14.3 (13.4–15.2)	93	5.7 (4.7–6.9)
10+ days	3718	37.1 (36.2–38.1)	424	16.0 (14.7–17.4)	2008	36.1 (34.8–37.4)	244	15.0 (13.3–16.8)
Cognitive spectrum disorder	3854	38.5 (37.5–39.4)		Not assessed	1985	35.6 (34.4–36.9)		Not assessed
Any known dementia	1735	17.3 (16.6–18.1)			833	15.0 (14.1–16.0)		
Known dementia alone (no delirium)	940	9.4 (8.8–10.0)			444	8.0 (7.3–8.7)		
Delirium superimposed on known dementia^a^	795	7.9 (7.4–8.5)			389	7.0 (6.3–7.7)		
Any delirium	2463	24.6 (23.8–25.5)			1290	23.2 (22.1–24.3)		
Full syndrome delirium (CAM+)	765	7.6 (7.1–8.2)			398	7.2 (6.6–7.9)		
Clinical history suggestive of delirium only	1698	17.0 (16.2–17.7)			892	16.0 (15.1–17.0)		
Delirium alone (no dementia)	1668	16.7 (16.0–17.4)			901	16.2 (15.2–17.2)		
Unspecified cognitive impairment^b^	451	4.5 (4.1–4.9)			251	4.5 (4.0–5.1)		

^a^These patients are also included in ‘any known dementia’ and ‘any delirium’

^b^AMT score < 8, no delirium, no known dementia

*CAM* Confusion Assessment Method, *OPRAA* Older Persons Routine Acute Assessment

One or more CSDs were present in 38.5% (95% CI 37.5–39.4) of admissions with an OPRAA assessment (Table 1). A known diagnosis of dementia was present in 17.3% (95% CI 16.6–18.1) of admissions, and 24.6% (95% CI 23.8–25.5) of admissions were for a person with delirium. Delirium superimposed on dementia was present in 7.9% (95% CI 7.4–8.5) of admissions (45.8% of people with known dementia had delirium; 32.3% of people with delirium had known dementia). A further 4.5% (95% CI 4.1–4.9) of admissions were for people with unspecified cognitive impairment (AMT < 8 in the absence of delirium or known dementia), most likely due to the presence of undiagnosed dementia.

### Incident cohort

**Fig. 1:**
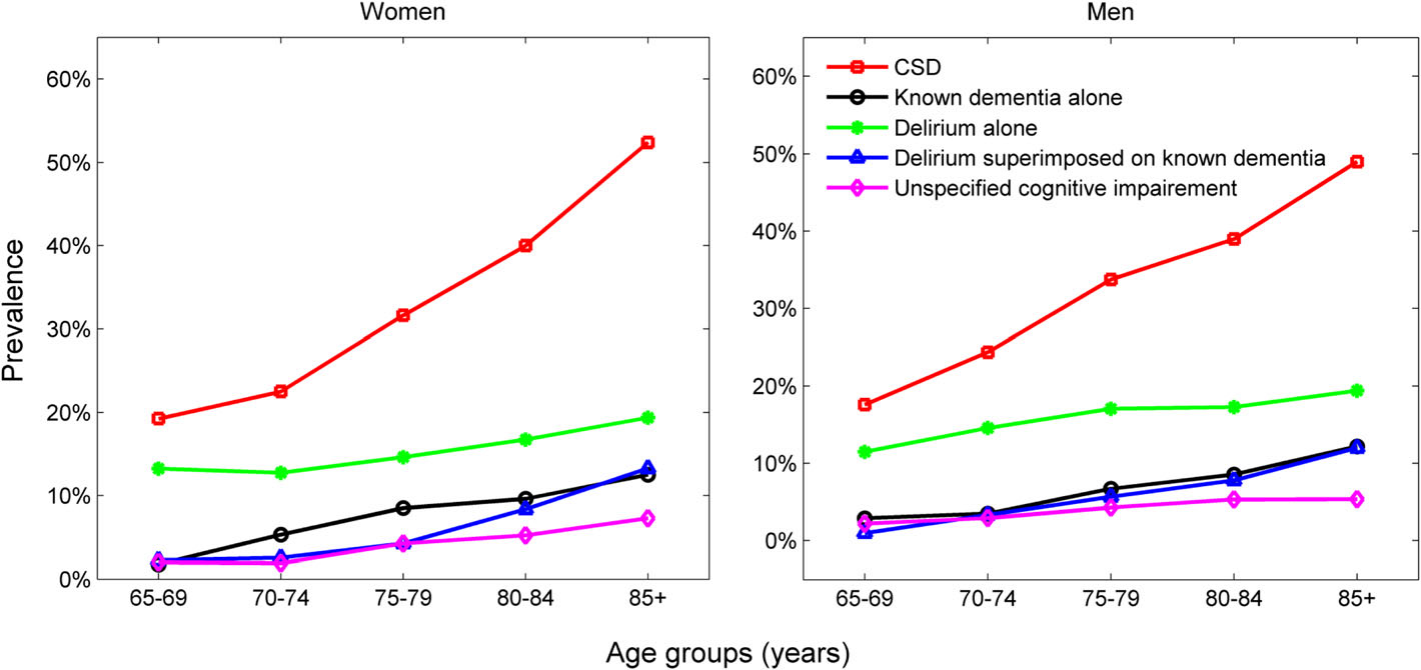
Prevalence of cognitive spectrum disorders by age and sex

Figure 1 shows the prevalence of CSDs in the incident cohort (n = 7201) by age and sex (detailed data is in Additional file 1: Table S1). Age was strongly associated with the prevalence of any CSD (Wald χ^2^ *P* < 0.001), rising from 18.4% in people aged 65–69 to 51.2% in people aged 85 years and over. After adjustment for age, CSD prevalence was not significantly associated with sex (Wald χ^2^ *P* = 0.776) and there was no evidence of an age-sex interaction (Wald χ^2^ *P* = 0.572), indicating that men and women share the same trend in CSD prevalence within the different age groups. Similar results were found for underlying mutually exclusive conditions (known dementia alone, delirium alone, delirium superimposed on known dementia and unspecified cognitive impairment).

### Outcome data in the incident cohort

Mean LOS in the incident cohort was 14.3 days (95% CI 13.6–15.0), and was longer in patients with a CSD (25.0 days, 95% CI 23.1–26.9) compared to those without CSD (11.8 days, 95% CI 11.0–12.6) (Table 2). Patients with delirium superimposed on dementia had significantly longer LOS (34.3 days, 95% CI 28.5–31.2) than those with dementia alone (20.1 days, 95% CI 16.9–23.3) or delirium alone (23.0 days, 95% CI 20.3–25.6).

**Table 2:** In hospital length of stay, 30-day mortality, and 1-year mortality by cognitive status

	In-hospital length of stay Mean days (95% CI)	Mortality by 30 days from admission Prevalence (95% CI)	Mortality by 1 year from admission Prevalence (95% CI)
All incident admissions (n = 7201)	14.3 (13.6–15.0)	9.9 (9.2–10.6)	29.0 (28.0–30.1)
Did not receive OPRAA assessment (n = 1632)	6.7 (5.6–7.8)^b^	7.1 (6.0–8.5)^c^	22.2 (20.3–24.3)^d^
Received OPRAA assessment (n = 5569)	16.8 (15.6–17.4)	10.7 (9.9–11.5)	31.0 (29.8–32.2)
No cognitive spectrum disorder (n = 3584)	11.8 (11.0–12.6)^e^	9.0 (8.1–10.0)^f^	26.0 (24.6–27.5)^g^
Cognitive spectrum disorder (n = 1985)	25.0 (23.1–26.9)	13.6 (12.2–15.2)	40.0 (37.9–42.2)
Known dementia alone (group 1, n = 444)	20.1 (16.9–23.3)^h^	12.8 (10.0–16.2)^i^	43.9 (39.4–48.5)^j^
Delirium alone (group 2, n = 901)	23.0 (20.3–25.6)	13.6 (11.5–16.0)	36.2 (33.1–39.4)
Delirium superimposed on known dementia (group 3, n = 389)	34.3 (28.5–40.0)	14.7 (11.5–18.5)	43.7 (38.9–48.7)
Unspecified cognitive impairment^a^ (group 4, n = 251)	26.8 (22.5–31.2)	13.2 (9.5–17.9)	41.0 (35.1–47.2)

^a^AMT score < 8, no delirium, no known dementia

^b^Difference = 9.8 (95% CI 8.1–11.6) *P* < 0.001

^c^Difference = 3.4 (95% CI 1.8–4.8) *P* < 0.001

^d^Difference = 8.8 (95% CI 6.4–11.1) *P* < 0.001

^e^Difference = 13.2 (95% CI 11.2–15.3) *P* <0.001

^f^Difference = 4.6 (95% CI 2.8–6.4) *P* <0.001

^g^Difference = 14.0 (95% CI 11.4–16.6) *P* < 0.001

^h^Group 3 significantly different from group 1 (*P* < 0.001) and group 2 (*P* < 0.001), all other pairwise comparisons not significant (*P* > 0.138)

^i^Pairwise comparisons not significant (*P* > 0.872)

^j^Group 1 significantly different from group 2 (*P* = 0.032) all other pairwise comparisons not significant (*P* > 0.054)

*OPRAA* Older Persons Routine Acute Assessment

Mortality is reported from date of admission in Table 2, but also from date of discharge combined with readmission in Table 3, in which just those individuals who are discharged alive were considered.

**Table 3:** Readmission or death by 30 days and by 1 year after discharge by cognitive status^a^

	Readmission or death by 30 days from discharge Prevalence (95% CI)	Readmission or death by 1 year from discharge Prevalence (95% CI)
All incident admissions discharged alive (n = 6465)	17.9 (17.0–18.9)	53.0 (51.8–54.2)
Did not receive OPRAA assessment (n = 1530)	15.4 (13.7–17.3)^c^	45.8 (43.3–48.3)^d^
Received OPRAA assessment (n = 4935)	18.7 (17.6–19.8)	55.2 (53.8–56.6)
No cognitive spectrum disorder (n = 3260)	17.4 (16.1–18.7)^e^	51.5 (49.8–53.2)^f^
Cognitive spectrum disorder (n = 1675)	21.2 (19.3–23.2)	62.4 (60.0–64.7)
Dementia no delirium (group 1 n = 385)	23.9 (19.9–28.4)^g^	68.3 (63.5–72.8)^h^
Delirium no dementia (group 2 n = 752)	20.9 (18.1–24.0)	57.4 (53.9–60.9)
Delirium superimposed on dementia (group 3 n = 327)	19.3 (15.4–23.9)	64.8 (59.5–69.8)
Unspecified cognitive impairment^b^ (group 4 n = 211)	20.4 (15.5–26.3)	65.4 (58.8–71.5)

^a^Excludes in hospital mortality (n = 736) as only patients discharged alive are included

^b^AMT score <8, no delirium, no known dementia

^c^Difference = 3.3 (95%CI 1.1-5.3) *P* = 0.003

^d^Difference = 9.4 (95%CI 6.6-12.3) *P* < 0.001

^e^Difference = 3.8 (95%CI 1.5-6.2) *P* = 0.001

^f^Difference = 10.9 (95%CI 8.0-13.8) *P* < 0.001

^g^Pairwise comparisons not significant (*P* > 0.444);

^h^Group 1 significantly different from group 2 (*P* = 0.002), all other pairwise comparisons not significant (*P* > 0.106)

*OPRAA* Older Persons Routine Acute Assessment

Mortality in the entire incident cohort was high, with 9.9% (95% CI 9.2–10.9) dying within 30 days of admission and rising to 29.0% (95% CI 28.0–30.1) at 1 year (Table 2). Patients with a CSD had higher mortality at 30 days after admission (13.6% vs. 9.0%, difference 4.6%, 95% CI 2.9–6.4) and at 1 year (40.0% vs. 26.0%, difference 14.0%, 95% CI 11.4–16.6). There was no clear pattern of varying mortality across different CSDs, although patients with dementia alone had significantly higher mortality at 1 year than those with delirium alone (Table 2).

Table 3 shows the prevalence of death or readmission after discharge alive following the incident admission. For all patients in the incident cohort, death or readmission occurred in 17.9% (95% CI 17.0–18.9) within 30 days of discharge, rising to 53.0% (95% CI 51.8–54.2) at 1 year. People with a CSD had significantly higher rates of death or readmission at 30 days and 1 year after discharge compared to those without (62.4% vs. 51.4%, difference 10.9%, 95% CI 8.0–13.8 at 1 year). Death or readmission by 30 days and 1 year after discharge showed no clear pattern across different CSDs, although patients with dementia alone had significantly higher mortality or readmission than those with delirium alone at 1 year after discharge.

**Fig. 2:**
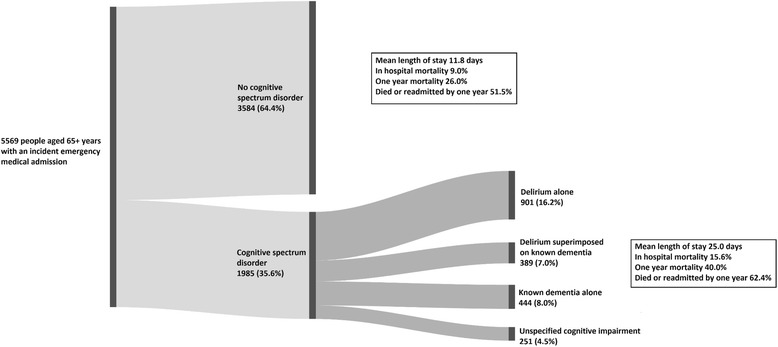
Prevalence and outcomes of incident emergency medical admissions with one or more cognitive spectrum disorder

Overall, LOS, mortality and readmission were statistically and clinically significantly worse for admitted older people with a CSD, but were high even for those with normal cognition (Fig. 2).

## Discussion

### Principle findings

In this study, over one-third of admissions in those aged 65 and over were for patients with a CSD, most commonly delirium (in 24.6% of all admissions) either on its own (16.7%) or superimposed on dementia (7.9%). Known dementia was less common than delirium (17.3% of all admissions) and almost half of admissions for people with known dementia were complicated by superimposed delirium. There were additionally 4.5% of admissions where there was unspecified cognitive impairment, in who many were likely to have undiagnosed dementia and therefore warranted post-discharge follow-up. As expected, the prevalence of CSD rose steeply with age, and CSD of some kind was present in half of admissions for patients aged 85 years and over. Older people with CSD had significantly worse outcomes than those without – mean LOS was 13.2 days longer, they had higher mortality in the year after admission (40.0% vs. 26.0%), and higher mortality or readmission in the year after discharge (62.4% vs. 51.5%). All categories of CSD were associated with poor outcomes, although LOS was greatest in those with delirium superimposed on dementia, and once discharged, patients with dementia alone had a higher mortality/risk of readmission or death than those with delirium alone.

### Strengths and weaknesses

The study’s strengths lie in the fact that it examines an unselected population cohort of older people admitted as a medical emergency to a single healthcare system (one acute hospital and four non-acute hospitals) providing all such care to patients resident in its catchment area, with complete follow-up of mortality and readmission outcomes using linked data from routine clinical practice. Although it is difficult to be precise about the generalisability of the findings, given the proportion of the population covered (7% of the Scottish population), the characteristics of the ageing population, and the standard mode of emergency admission into non-specialised acute hospital care in the UK, parts of Europe, North and South America, and Australasia, it is assumed that, due to the large sample size and time period covered, these findings will not be dissimilar to other parts of the world where this healthcare system exists.

The study reports on the findings of a brief standardised screening assessment of all emergency admissions. Analysis is not restricted to specific types of cognitive disorder, which is important given the overlaps and the poor outcomes observed across all CSD groups, and therefore individual conditions such as dementia or delirium are viewed in the context of all cognitive disorder subtypes. Additionally, when discussing the strengths of the work, the size of the population examined is notable. By using routine data, the study included 12,673 emergency medical admissions in 8374 patients, which is more than the total patients in all studies included in the most recent systematic reviews of dementia [7] and delirium [8] in hospital inpatients.

The key limitations and possible sources of bias reflect the use of routine healthcare data and the cross-sectional nature of the OPRAA assessment. The OPRAA assessment was introduced to support the initial multi-disciplinary assessment and management of frail older patients as part of a clinical service. This raises five areas that require further discussion, namely (1) coverage, (2) accuracy of brief assessment tools, (3) cross-sectional nature of assessment, (4) lack of full dementia diagnostic workup, and (5) differences between admission and incident cohorts.

1. Coverage
  By design, the OPRAA assessment was not performed in patients with brief admissions to exclude serious illness like myocardial infarction in people with chest pain, or who required immediate escalation to critical care, or who were admitted for palliative care. OPRAA coverage was therefore 79.0% of all admissions and 77.3% of incident admissions. However, this compares favourably with most consented research cohorts, including those with the highest coverage, such as Sampson et al. [14], who in their study of dementia prevalence screened 88.2% of people aged 70 years and older admitted for at least 48 hours, and included 76.7% (617 patients in total) after exclusions. For comparison, 88.3% of all admissions of over 48 hours in those aged above 70 were included in this analysis.
2. Accuracy of brief assessment tools
  OPRAA used relatively simple instruments suitable for identifying delirium and cognitive impairment in a routine clinical context, which may not always match assessment using gold-standard research instruments, although OPRAA assessment was performed by trained, experienced specialist nurses. The sensitivities of the screening tools used in OPRAA have been discussed in the literature.
  Only 31% of people diagnosed with delirium in this dataset were CAM positive. This contrasts with the literature comparing CAM to a gold-standard assessment of delirium, where CAM sensitivity ranges from 46% to 100% [27]. This likely reflects the difference between assessments done by dedicated staff during research studies and assessments like OPRAA performed in routine clinical practice where high workload and competing clinical demands constrain when assessments can be done, making it difficult to repeatedly return to perform an optimal assessment (for example, with an informant present). During the period of the study, the nurses applied the original scoring for the CAM in terms of CAM positivity requiring “an acute ‘and’ fluctuating course”, which the CAM developers have since recognised is often difficult to assess when using the CAM in routine clinical practice. The CAM manual was updated in 2014 to allow two methods of scoring this criteria [28]. It states that the original scoring (‘and’) maximises specificity but reduces sensitivity in clinical use, and suggests the use of “an acute ‘or’ fluctuating course” to maximise sensitivity at the cost of specificity. In addition, delirium by its nature is fluctuant, and others have found that CAM positivity varies over time in people with delirium, with, for example, 35% of assessments being CAM negative in people with hip fracture who were ever CAM positive [29]. As implemented in this study, CAM would therefore be expected to be highly specific but less sensitive, which is consistent with the observed patterns, and with the conclusion of a recent systematic review of the CAM that “*the use of these tools should not replace clinical judgement*” [30].
  Similar discussions are present for the AMT in the literature. Initial reports of the accuracy of the AMT in screening for cognitive impairment suggested “*The best cut-off point was 8, with less than 8 suggesting abnormal cognitive function*” [31]. A recent systematic review and meta-analysis examined its accuracy when used as an instrument to screen for dementia [32, 33]. In this meta-analysis with a cut-off of < 7, pooled analysis of the AMTs showed a sensitivity of 81%, and a specificity of 84% for a diagnosis of dementia. As noted in this paper a cut-off of < 8 is considered more usual in clinical practice. In the current study, we use a cut-off of < 8 to report unspecified cognitive impairment.
3. Cross-sectional nature of assessment
  The OPRAA assessment was carried out within the first 24 hours of admission, and therefore captures prevalent cases of CSD at time of admission. Any changes in patients’ cognitive status during the course of admission are not captured in the study design. For example, patients admitted to hospital with no CSD or with known dementia alone may develop incident delirium through the course of their admission, and their outcomes will be narrowing the divide between the CSD subgroups in the reported analyses.
4. Lack of full dementia diagnostic workup
  Data on results of further diagnostic workups for definitive diagnoses of dementia are not included. As such, the categories of the CSDs are based on the diagnoses that were known about at the time of admission, i.e. known dementia, along with diagnoses that can be attributed as a result of the brief assessment. It is therefore most likely that those patients with a low AMT (unspecified cognitive impairment) are those with undiagnosed dementia.
5. Differences between admission and incident cohorts
  For analysis, two cohorts were examined. Within the admission (prevalence) cohort each hospital episode is featured and therefore an individual may be counted a number of times with each readmission to hospital. The incident cohort differs from the admission cohort in that it identifies individuals at the beginning of their interaction with acute healthcare services and follows them through that journey capturing all re-admissions and mortality. Outcomes reported from this incident cohort are applicable therefore to individual patients. Data from the admissions (prevalence) cohort can be seen as reporting the impact that this population has on the acute hospital.
6. Lack of adjustment for other factors
  The analysis reported here is unadjusted for other factors which may be associated with the outcomes, including physical health, function and nutrition. The OPRAA assessment did not include evaluation of nutrition. It did include an assessment of Activities of Daily Living (ADL) and variation in function may explain some of the observed associations. This is an area that requires further in-depth analysis since declines in ADL may reflect both physical and/or cognitive impairment, making adjustment complicated, and any interaction between cognitive status and ADL may vary with time.

### Comparison with other studies

The estimated prevalence of dementia and delirium varies widely in the literature, reflecting varying age inclusion criteria in particular, and the specific diagnosis focused on and methods for its ascertainment. Reported dementia prevalence ranges from 2.8% to 63.0% [7] and delirium prevalence from 10.0% to 31.0% [8], compared with this study which found a prevalence of 17.3% for known dementia, 4.5% for likely undiagnosed dementia (low AMT with no known dementia and no delirium), and 24.6% for delirium. In particular, the methodological differences of age cut-off, minimum LOS plus subsequent diagnostic workup account for the reported differences in prevalence when comparison is made with the paper by Sampson et al. [14]. In Sampson’s cohort, patients recruited were aged 70 or over and had to have a LOS of 48 hours or more to be included. Their study also followed up those patients with unspecified cognitive impairment, who underwent a full dementia diagnostic workup. Prior to this workup their prevalence rate of known dementia was approximately 20%. When compared with the current study, the prevalence of known dementia (with and without delirium superimposed) in those patients aged 70 or over with a LOS of over 2 days is 18.3%, which is in agreement with the findings in the Sampson paper.

Additionally, the reporting of the prevalence of delirium superimposed on dementia frequently uses the population with known dementia as the reference population. This is the case in the review by Fick et al. [10], where the prevalence of delirium superimposed on dementia ranges from 22.0% to 89.0% of older inpatients with dementia. Once again, our finding of a prevalence of delirium of 45.8% of those with known dementia (7.9% of all admissions) compares favourably with previous findings.

Also worth noting is that most studies of dementia prevalence exclude those with delirium, but in this study almost one-third of those with delirium had known dementia, emphasising that a single condition focus may be misleading.

Only a limited number of previous studies have examined mortality or readmission, with some evidence of higher mortality in people with dementia [32, 33], but conflicting findings in other studies [13, 34]. Higher mortality is reported for delirium [8, 9, 35–37], delirium superimposed on dementia [10, 15, 38], and cognitive impairment irrespective of cause [11]. A striking feature of this study is that outcomes (LOS, mortality after admission, and death or readmission after discharge) are significantly worse in people with any CSD with relatively little difference between different types of CSD, although delirium superimposed on dementia was associated with significantly longer LOS compared to delirium or dementia alone, and dementia alone was associated with significantly higher mortality compared to delirium alone.

### Implications of the study

This study shows that over one-third of emergency medical admissions in people aged 65 years and over will be for individuals with a CSD, who will stay in hospital on average almost 2 weeks longer than those without. Almost one in seven of those with CSD will die in the 30 days after admission, and two fifths will die in the year after admission. Of those who survive to be discharged, one in five will die or be readmitted within 30 days, and three in five within a year. These findings are put in context in Box 1.

The key implication is that healthcare systems have to systematically identify and manage CSD in older people admitted as medical emergencies, but avoid only focusing on dementia or delirium alone. Additionally, those with likely undiagnosed dementia (low AMT without known dementia or delirium) need follow-up for diagnosis after the acute episode. Condition-specific care plans/pathways such as those for dementia or delirium alone risk missing the complexities of a person-centred approach to CSDs. There is good evidence of reduced mortality and nursing home admission after discharge from ‘comprehensive geriatric assessment’ of older inpatients, which includes co-ordinated multidisciplinary assessment, geriatric medicine expertise, a problem- rather than a disease-focused approach [39], and the creation and implementation of a longer-term management plan.

### Future research implications

Further longitudinal research and analysis adjusting for physical comorbidity and function is needed to examine whether cognitive impairment is an independent predictor of mortality or whether worse outcome is mediated by physical comorbidity, by functional status or frailty. The very high mortality observed in people with CSD (40.0% by 1 year after admission) requires further examination, since it is likely a mixture of both unavoidable deaths and inadequate management of older people with cognitive impairment (although it is also important to note that mortality is also high in those without CSD – 26% by 1 year). Research is needed to identify how best to distinguish which older inpatients would be better served by a palliative approach to care and which require the same high-intensity diagnosis and management as younger people, and to develop and evaluate interventions to ensure appropriate delivery of both.

## Conclusions

Over one-third of admissions to hospital in the older population have a CSD, and this is associated with worse outcomes. Delirium is more common than dementia in the acute hospital, but of those people with known dementia, 46% had delirium superimposed. There is significant overlap between all these conditions with outcomes that are broadly similar across the spectrum of disorders. Management pathways should aim to be person focused and encompass the spectrum of these disorders rather than condition specific. Further research is needed to determine direct causal relationships, predictors of decline and optimal care pathways for this very common, vulnerable and complex population.

## Box 1 What this paper adds

***What is already known on this subject***

In the hospital setting, cognitive impairment may be due to a number of overlapping conditions – people may have pre-existing dementia, may have developed delirium, may have delirium superimposed on dementia, or may have unspecified cognitive impairment due to undiagnosed dementia, adverse effects of medication, poorly controlled physical morbidities (e.g. diabetes) or a combination of these.

Research to date in older people admitted to hospital has studied the epidemiology and outcomes of these conditions in isolation.

This study examines the relative frequencies and associated outcomes of people with the full range of cognitive spectrum disorders (CSDs) in a large, unselected population of older people admitted as an acute medical emergency.

***What this study adds***

The results show that cognitive spectrum disorders (delirium, dementia, delirium superimposed on dementia, or non-specified cognitive impairment) affect over one-third of older acute medical admissions and over a half of those over the age of 85 years. Delirium is most common (24.6%) followed by known dementia (17.3%), with 7.9% of those with dementia having delirium superimposed.

Patients with CSDs have an excess length of stay of 13.2 days, have increased mortality (40.0% vs. 26.0% at 1 year after admission), and increased risk of death or readmission once discharged (59.4% vs. 49.4% at 1 year).

The key implication is that healthcare systems should systematically identify and manage CSD in older people admitted as medical emergencies, but avoid only focusing on dementia or delirium alone. Additionally, they should ensure that those with likely undiagnosed dementia (low Abbreviated Mental Test score without known dementia or delirium) are followed up. At practice level, this necessitates the design of care pathways for patients with CSD as opposed to condition-specific management. Further longitudinal research is needed to examine whether cognitive impairment is an independent predictor of mortality or whether worse outcome is mediated by physical comorbidity, functional status, frailty or people with cognitive impairment receiving less effective care

## Additional file

Additional file 1: Table S1. Cognitive spectrum disorder prevalence broken down by sex and age groups. (DOC 42 kb)

## Abbreviations

ADL: Activities of daily living
AMT: Abbreviated Mental Test
AMU: Acute medical unit
CAM: Confusion Assessment Method
CHI: Community Health Index
CSD: cognitive spectrum disorder
HIC: Health Informatics Centre
LOS: length of stay
OPRAA: Older Persons Routine Acute Assessment
SMR: Scottish Morbidity Records
SOPs: standard operating procedures
