# Supplementary material for: Epidemiology and outcomes of people with dementia, delirium, and unspecified cognitive impairment in the general hospital: prospective cohort study of 10,014 admissions
Source: BMC Med. 2017 Jul 27;15:140. doi: 10.1186/s12916-017-0899-0 (PMC5530485; doi:10.1186/s12916-017-0899-0)
Supplement: Additional file 1: Table S1. — Cognitive spectrum disorder prevalence broken down by sex and age groups. (DOC 42 kb) [file 12916_2017_899_MOESM1_ESM.doc]

**Table S1** Cognitive spectrum disorder prevalence broken down by sex and age groups

|  | Cognitive spectrum disorder Prevalence (95% CI) | Known dementia alone Prevalence (95% CI) | Delirium alone Prevalence (95% CI) | Delirium superimposed on known dementia Prevalence (95% CI) | Unspecified cognitive impairment Prevalence (95% CI) |
| --- | --- | --- | --- | --- | --- |
| All patients (n = 5569) | 35.6 (34.4–36.9) | 8.0 (7.3–8.7) | 16.2 (15.2–17.2) | 7.0 (6.3–7.7) | 4.5 (4.0–5.1) |
| Women | | | | | |
| All patients (n = 3121) | 37.2 (35.5–38.9) | 8.7 (7.7–9.7) | 16.2 (14.9–17.5) | 7.5 (6.7–8.5) | 4.8 (4.1–5.6) |
| 65–69 (n = 400) | 19.3 (15.7–23.4) | 1.8 (0.9–3.6) | 13.3 (10.3–16.9) | 2.3 (1.2–4.2) | 2.0 (1.0–3.9) |
| 70–74 (n = 471) | 22.5 (19.0–26.5) | 5.3 (3.6–7.7) | 12.7 (10.0–16.1) | 2.6 (1.5–4.4) | 1.9 (1.0–3.6) |
| 75–79 (n = 588) | 31.6 (28.0–35.5) | 8.5 (6.5–11.0) | 14.6 (12.0–17.7) | 4.3 (2.9–6.2) | 4.3 (2.9–6.2) |
| 80–-84 (n = 645) | 40.0 (36.3–43.8) | 9.6 (7.6–12.1) | 16.7 (14.1–19.8) | 8.4 (6.5–10.8) | 5.2 (3.8–7.2) |
| 85+ (n = 1017) | 52.4 (49.3–55.5) | 12.5 (10.6–14.7) | 19.4 (17.1–21.9) | 13.3 (11.3–15.5) | 7.3 (5.8–9.0) |
| Men | | | | | |
| All patients (n = 2448) | 33.7 (31.9–35.6) | 7.1 (6.1–8.2) | 16.2 (14.8–17.7) | 6.3 (5.4–7.3) | 4.1 (3.4–5.0) |
| 65–69 (n = 409) | 17.6 (14.2–21.6) | 2.9 (1.7–5.0) | 11.5 (8.8–14.9) | 1.0 (0.4–2.5) | 2.2 (1.2–4.1) |
| 70–74 (n = 480) | 24,4 (20.8–28.4) | 3.5 (2.2–5.5) | 14.6 (11.7–18.0) | 3.3 (2.1–5.3) | 2.9 (1.7–4.8) |
| 75–79 (n = 492) | 33.7 (29.7–38.0) | 6.7 (4.8–9.3) | 17.1 (14.0–20.6) | 5.7 (4.0–8.1) | 4.3 (2.8–6.4) |
| 80–84 (n = 526) | 39.0 (34.9–43.2) | 8.6 (6.5–11.3) | 17.3 (14.3–20.8) | 7.8 (5.8–10.4) | 5.3 (3.7–7.6) |
| 85+ (n = 541) | 49.0 (44.8–53.2) | 12.2 (9.7–15.2) | 19.4 (16.3–23.0) | 12.0 (9.5–15.0) | 5.4 (3.8–7.6) |
| Logistic regression | | | | | |
| Sex | 0.776 | 0.676 | | | |
| Age | <0.001 | <0.001 | | | |
| Sex*Age | 0.572 | 0.500 | | | |
